# Cost-Effectiveness of Posaconazole vs. First-Generation Triazoles for the Prevention of Invasive Fungal Infections Among High-Risk Patients With Hematological Malignancies in China

**DOI:** 10.3389/fpubh.2022.884846

**Published:** 2022-05-17

**Authors:** Changcheng Shi, Jian Ye, Yaping Xie, Rong Dong, Weizhong Jin, Linling Wang, Yingying Fang, Qiyuan Shan, Nengming Lin

**Affiliations:** ^1^Department of Clinical Pharmacy, Key Laboratory of Clinical Cancer Pharmacology and Toxicology Research of Zhejiang Province, Affiliated Hangzhou First People's Hospital, Zhejiang University School of Medicine, Hangzhou, China; ^2^Department of Respiratory Medicine, Affiliated Hangzhou First People's Hospital, Zhejiang University School of Medicine, Hangzhou, China; ^3^Department of Hematology, Affiliated Hangzhou First People's Hospital, Zhejiang University School of Medicine, Hangzhou, China; ^4^Department of Pharmacy, Shaoxing Hospital of Traditional Chinese Medicine, Shaoxing, China; ^5^Department of Pharmacy, Hangzhou Cancer Hospital, Hangzhou, China; ^6^School of Pharmaceutical Sciences, Zhejiang Chinese Medical University, Hangzhou, China

**Keywords:** cost-effectiveness, posaconazole, fluconazole, itraconazole, invasive fungal infections, hematological malignancies

## Abstract

**Background:**

Posaconazole is confirmed to be more effective for preventing invasive fungal infections (IFIs) than first-generation triazoles (fluconazole and itraconazole), but its economic value has not been comprehensively evaluated in China. This study compared the cost-effectiveness of these two antifungal prophylaxis regimens in hematological-malignancy patients at high risk for IFIs from the Chinese healthcare perspective.

**Methods:**

A hybrid decision tree and Markov model were built using published data to estimate the total costs and quality-adjusted life-years (QALYs) of antifungal prophylaxis with posaconazole oral suspension and first-generation triazoles. Regimens with an incremental cost-effectiveness ratio (ICER) lower than the threshold of willingness to pay (WTP) were considered cost-effective. One-way and probabilistic sensitivity analyses were performed to assess model robustness. The regional imbalance of economic development and the tablet formulation of posaconazole were considered in the scenario analyses.

**Results:**

In the base-case analysis, posaconazole oral suspension provided an additional 0.109 QALYs at an incremental cost of $954.7, yielding an ICER of $8,784.4/QALY, below the national WTP threshold of $31,315/QALY. One-way and probabilistic sensitivity analyses showed that the results were robust. Scenario analyses showed that the base-case ICER was consistently below the WTP thresholds of all 31 Chinese provinces, with the likelihood of posaconazole being cost-effectiveness ranging from 78.1 to 99.0%. When the posaconazole oral suspension was replaced by the tablet formulation, the ICER increased to $29,214.1/QALY, still below the national WTP threshold and WTP thresholds of 12 provinces.

**Conclusions:**

Posaconazole oral suspension is a highly cost-effective regimen for preventing IFI in high-risk hematological-malignancy patients from the Chinese healthcare perspective. Posaconazole tablets may also be considered in some high-income regions of China.

## Introduction

Invasive fungal infection (IFI) remains a serious complication in hematological-malignancy patients and is associated with substantial morbidity and mortality. In China, the incidence of proven or probable IFI was estimated at a rate of 2.1% per chemotherapy course, with a death rate of 11.7% according to data from the China Assessment of Antifungal Therapy in Hematological Diseases (CAESAR) study, which prospectively enrolled 4,192 patients undergoing chemotherapy for hematological malignancies at 35 Chinese hospitals (1). Patients with IFIs have been reported to have significantly increased hospitalization costs, which impose a heavy economic burden on the healthcare system worldwide (2, 3).

It is well known that IFIs are difficult to diagnose and manage (4, 5). Antifungal prophylaxis for high-risk populations, especially those with acute myelogenous leukemia (AML) and myelodysplastic syndrome (MDS) who are undergoing chemotherapy, is reasonable and has been widely recommended as a standard strategy in clinical practice (6–9). Posaconazole, a second-generation triazole with a broad antifungal spectrum, has been strongly recommended in multiple foreign guidelines as the sole first-line agent with the highest level of evidence and recommendation for IFI prevention in high-risk AML or MDS patients (6–8). These recommendations were initially built on the basis of a pivotal randomized controlled trial (RCT), which showed a lower IFI occurrence rate and longer survival time in hematological malignancy patients receiving posaconazole than in those receiving the first-generation triazoles fluconazole or itraconazole (10). More recently, several network meta-analyses of RCTs (11–13) further confirmed that posaconazole prevented IFI more effectively and yielded a higher survival rate than comparator antifungal agents, highlighting its benefits in IFI prophylaxis.

Posaconazole was initially marketed as an oral suspension in China in 2013. Almost a decade later, its prophylactic use is still limited in Chinese patients with hematological malignancies, although it is preferred in terms of clinical benefits. Data from the CAESAR study showed that the top two most frequently used antifungal agents for IFI prophylaxis in China were the first-generation triazoles fluconazole and itraconazole, accounting for 48.4 and 26.0% of triazoles, respectively (1). Notably, a recent survey in seven Asian countries reported that for 80% of respondents, the most common reason for not using the preferred antifungal agent was the cost (14). As an innovative antifungal agent, the price of posaconazole in China remains high. Although posaconazole is confirmed to be more effective for preventing IFIs than first-generation triazoles, its economic value has not been comprehensively evaluated in China. Owing to scarce healthcare resources in China, the widespread use of posaconazole should be comprehensively evaluated by balancing clinical outcomes and expenditures.

The primary aim of this study was to compare the cost-effectiveness of posaconazole oral suspension vs. first-generation triazoles in IFI prevention in high-risk patients with hematological malignancies from the Chinese healthcare perspective. As a tablet formulation of posaconazole has also been available in China since 2018, an economic evaluation of posaconazole tablets was also performed in this study.

## Methods

### Model Design

**Figure 1:**
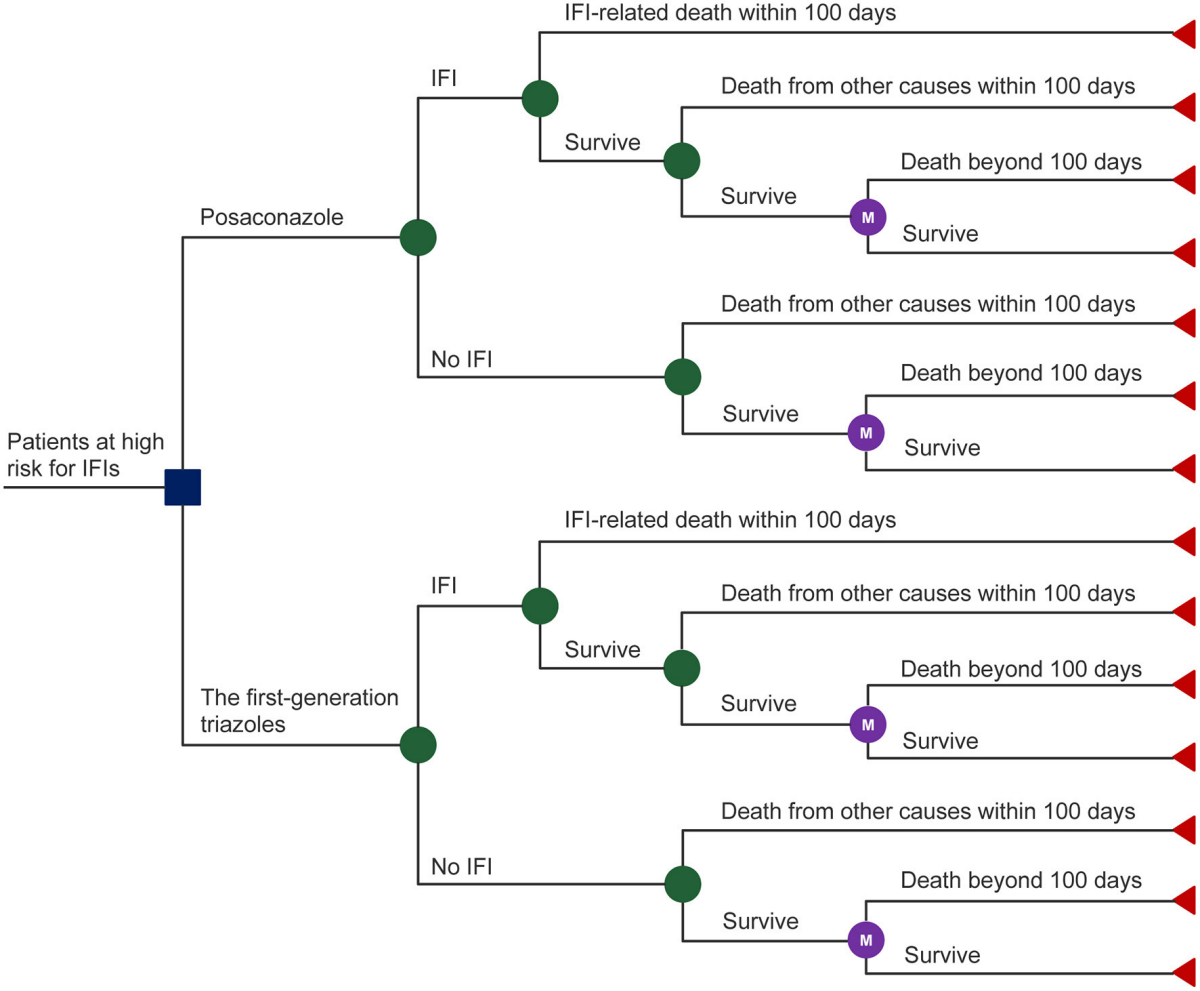
Structure of the model for analysis.

A mathematical model was adopted to evaluate the cost-effectiveness of posaconazole vs. first-generation triazoles for IFI prevention in high-risk hematological malignancy patients. The model comprised two components (Figure 1): (i) a decision tree model that corresponded to the initial 100-day outcomes after prophylactic use of antifungal agents based on the pivotal RCT (10). At the end of the initial 100-day period, patients may die from IFIs or from other causes or may survive with or without IFIs. (ii) a lifetime Markov model that represents the outcomes of the patients who survive beyond the initial 100-day period. A one-month Markov cycle length was applied with half-cycle correction. Total costs and quality-adjusted life-years (QALYs) were estimated to calculate the incremental cost-effectiveness ratio (ICER). The model was built and analyzed using Treeage Pro Healthcare (Version 2022 R 1.0, Williamstown, MA, USA).

### Study Population

The hypothetical patient characteristics were in line with those reported in a pivotal RCT (10). Briefly, the trial included a total of 602 patients with hematological malignancies from 89 centers worldwide. Of these, 86% (515/602) had AML, and the remaining 14% (87/602) had MDS. These AML or MDS patients were randomly assigned to receive posaconazole oral suspension (*n* = 304) or first-generation triazoles (*n* = 298). In the first-generation triazole group, approximately 81% of patients received fluconazole and the remaining patients received itraconazole. The dose and duration of antifungal prophylaxis regimens were obtained from the pivotal RCT as follows: posaconazole oral suspension (200 mg three times daily for 29 days), fluconazole (400 mg once daily for 24 days) and itraconazole (200 mg twice daily for 29 days). The mean age was approximately 50 years, and the proportion of females was 47.2% (10).

### Clinical and Utility Data

The probability of IFI occurrence after antifungal prophylaxis, IFI-related mortality, and mortality from other causes during the initial 100-day period were taken from a pivotal RCT (10). In the pivotal RCT, the probability of IFI occurrence with posaconazole was significantly lower (4.6%, 14/304) than that with first-generation triazoles (11%, 33/298). The IFI-related mortality was assumed to be equal for each group and was pooled for analysis, although this parameter was lower numerically in the posaconazole group (35.7%, 5/14) than in the first-generation triazole group (48.5%, 16/33). The same assumption and data processing were applied to the term of the mortality from other causes not attributable to IFI.

The probabilities of 5-year survival for AML and MDS were obtained from published data (15, 16). In a previous systematic review focusing on the health state utility values for AML, the reported utility estimates for individuals undergoing induction chemotherapy were between 0.524 and 0.670, while utility estimates for individuals in remission post-chemotherapy were between 0.81 and 0.91 (17). As recommended by the authors, we considered utility values of 0.648 and 0.830 for AML patients in the initial 100-day period (induction chemotherapy phase) and the subsequent Markov cycles (post chemotherapy remission phase), respectively. Because of the relatively high proportions of AML patients (86%) included in the pivotal RCT (10) and the limited utility data for MDS patients in the current study, AML utilities were assumed to represent the utilities of the target populations in the model analysis. The benefits were discounted at 5% annually (18). The key clinical and utility inputs are presented in Table 1.

**Table 1:** Input parameters for model analyses.

**Model parameter**	**Base case**	**Range**	**Distribution**	**References**
Probability of IFI with posaconazole	0.046	0.034–0.058	Beta	(10)
Probability of IFI with the first-generation triazoles	0.111	0.083–0.139	Beta	(10)
Probability of IFI-related mortality	0.447	0.335–0.559	Beta	(10)
Probability of mortality from other causes	0.158	0.119–0.198	Beta	(10)
5-year relative survival for AML patients	0.44	0.33–0.55	Uniform	(15)
5-year relative survival for MDS patients	0.52	0.39–0.65	Uniform	(16)
IFI treatment costs ($)	5,423.3	4,067.5–6,779.1	Uniform	(20)
**Daily drug costs ($)**				
Posaconazole (oral suspension)	45.56	34.17–56.95	Uniform	(19)
Posaconazole (tablets)	119.98	89.99–149.98	Uniform	(19)
Fluconazole	0.26	0.20–0.33	Uniform	(19)
Itraconazole	1.89	1.42–2.36	Uniform	(19)
**Mean duration of prophylaxis (days)**				
Posaconazole	29	21.75–36.25	Gamma	(10)
Fluconazole	24	18.00–30.00	Gamma	(10)
Itraconazole	29	21.75–36.25	Gamma	(10)
Discount rate	5%	0-8%	Uniform	(18)
Utility for patients undergoing induction treatment	0.648	0.486–0.810	Uniform	(17)
Utility for patients in remission post-chemotherapy	0.830	0.623–1.000	Uniform	(17)

*AML, acute myelogenous leukemia; IFI, invasive fungal infection; MDS, myelodysplastic syndrome*.

### Cost Data

Only direct medical costs were calculated in the model analysis, which mainly included the drug acquisition costs and management of IFIs (Table 1). The frequency of adverse events in patients who received posaconazole and first-generation triazoles were similar and relatively low (10). In addition, the available evidence of its impact on costs was limited. Therefore, costs of adverse events were not included in our study. Costs for treatment of the primary disease (AML and MDS) were also not considered in this study, as they were regarded to be equivalent between the two prophylaxis regimens. The unit prices of antifungal agents were collected from the Yaozh database (19). Due to the availability of generic fluconazole and itraconazole, the lowest price of the generic agents was used in the analysis. The dose and duration of antifungal prophylaxis regimens were obtained from the pivotal RCT (10). In the first-generation triazole group, approximately 81% of patients received fluconazole and the remaining patients received itraconazole, which was in line with the distributions in the pivotal RCT (10). The management costs of IFIs were derived from an analysis using data from the CAESAR study (20). All costs were inflated to 2020 values and converted into United States dollars ($) by the exchange rate: $100 = ¥689.76 (21). Because all costs that should be calculated in the model were incurred in the initial 100 days after prophylaxis regimens were applied, the costs were not discounted in our analysis.

### Sensitivity Analysis

One-way deterministic sensitivity analysis (DSA) and probabilistic sensitivity analysis (PSA) were carried out to evaluate model robustness. In one-way DSA, input parameters were changed one-by-one within 95% confidence intervals (CIs) or a variance of 25% from their base-case values if 95% CIs were not available. Utility measurements were set at 1 if the base values plus 25% were >1. The results of one-way DSA are presented as a tornado diagram. In PSA, a Monte Carlo simulation of 1,000 iterations by varying all parameters simultaneously with a prespecified distributions was performed (22). The PSA results are presented as probabilistic scatter plots and cost-effectiveness acceptability curves. The threshold of willingness to pay (WTP) was set at three times the gross domestic product (GDP) per capita of China in 2020 ($31,315) (21).

### Scenario Analysis

Considering the imbalance of economic development among different provinces in China, the first scenario analysis was conducted to assess the probability that posaconazole prophylaxis is cost-effective when compared with first-generation triazoles under province-level WTP thresholds (three times the province-level GDP per capita) (21).

In the second scenario analysis, the oral suspension of posaconazole was replaced by the tablet formulation. The clinical efficacy for IFI prophylaxis and the duration of prophylaxis were assumed to be equal between the two oral posaconazole formulations. According to the package insert, those patients receiving posaconazole tablets were administered 300 mg twice daily on day 1 and then 300 mg once daily from day 2 onward. The unit price of posaconazole tablets was also collected from the Yaozh database (19).

## Results

### Base-Case Analysis

The results of the base-case analysis are presented in Table 2. In the first-generation triazole group, the total costs per patient were $616.3, of which 2.5% ($15.5) were drug costs for prophylactic use, and 97.5% ($600.8) were costs attributed to the management of IFI. The total cost per patient was estimated at $1,571 in the posaconazole group. Patient prophylaxis with posaconazole oral suspension showed higher drug acquisition costs ($1,321.2) but lower IFI management costs ($249.8) than patient prophylaxis with first-generation triazoles.

**Table 2:** Cost-effectiveness results of posaconazole vs. first-generation triazoles.

**Outcomes**	**First-generation** **triazoles**	**Posaconazole** ^**a**^	
		**Oral suspension**	**Tablets**
Total costs ($)	616.3	1,571.0	3,729.2
Drug costs for prophylaxis ($)	15.5	1,321.2	3,479.4
IFI management costs ($)	600.8	249.8	249.8
LYs	4.355	4.488	4.488
QALYs	3.574	3.683	3.683
Incremental costs ($)^a^	/	954.7	3,112.9
Incremental LYs^a^	/	0.132	0.132
Incremental QALYs^a^	/	0.109	0.109
ICER ($/LY)^a^	/	7,209.4	23,506.6
ICER ($/QALY)^a^	/	8,784.4	28,641.8

a *Compared to the reference regimen (the first-generation triazoles)*.

*ICER, incremental cost-effectiveness ratio; IFI, invasive fungal infection; LY, life-year; QALY, quality-adjusted life-year*.

The life expectancy of patients using the posaconazole oral suspension was 4.488 life-years (LYs), which was 0.132 LYs more than patients using the first-generation triazoles (4.355 LYs). Accounting for quality of life, patients in the posaconazole group gained 3.683 QALYs, which was 0.109 QALYs more than patients in the first-generation triazoles group (3.574 QALYs).

Based on the above data, prophylactic use of the posaconazole oral suspension had an ICER of $8,784.4/QALY compared to the first-generation triazoles, which was below the national WTP threshold ($31,315/QALY), indicating that the posaconazole oral suspension was more cost-effective than the first-generation triazoles.

### Sensitivity Analysis

**Figure 2:**
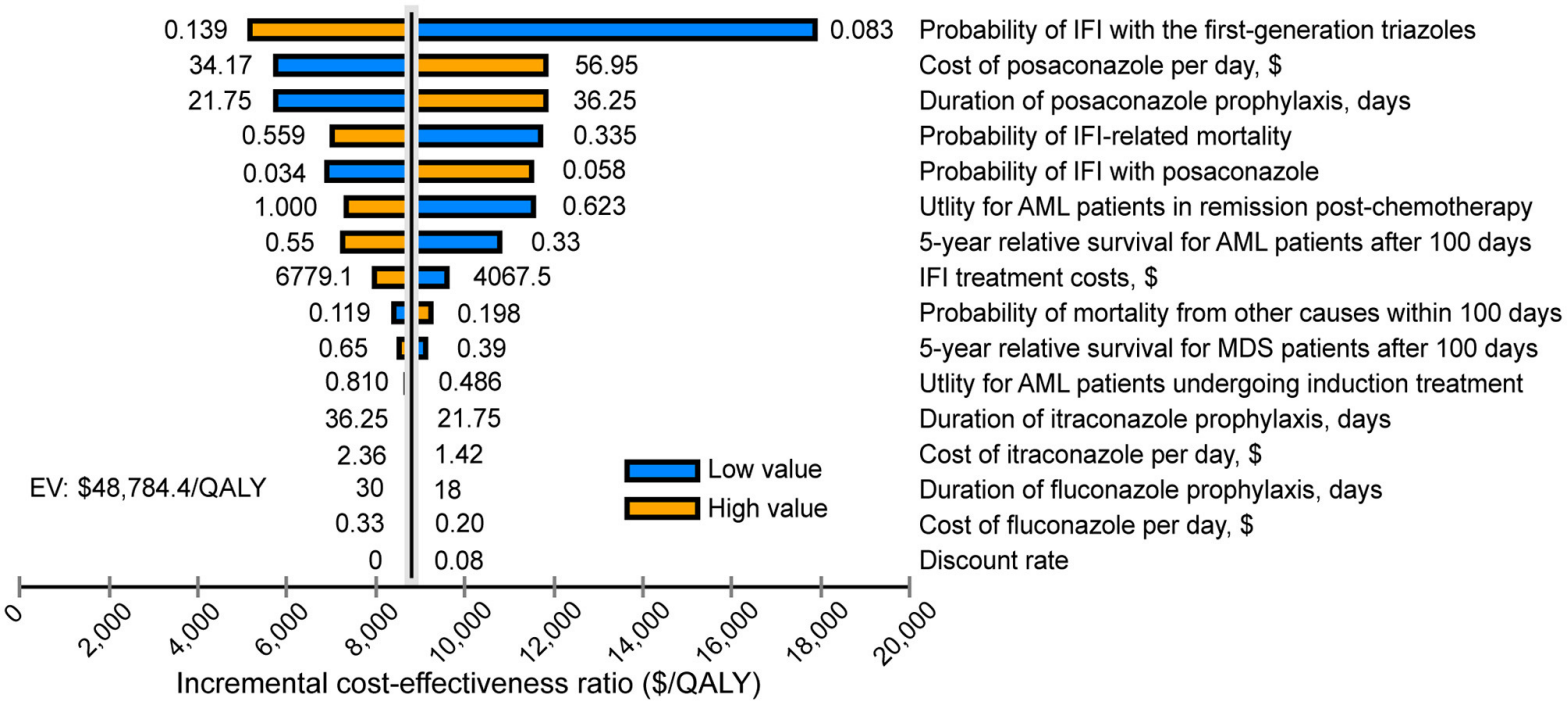
One-way sensitivity analysis tornado diagram.

**Figure 3:**
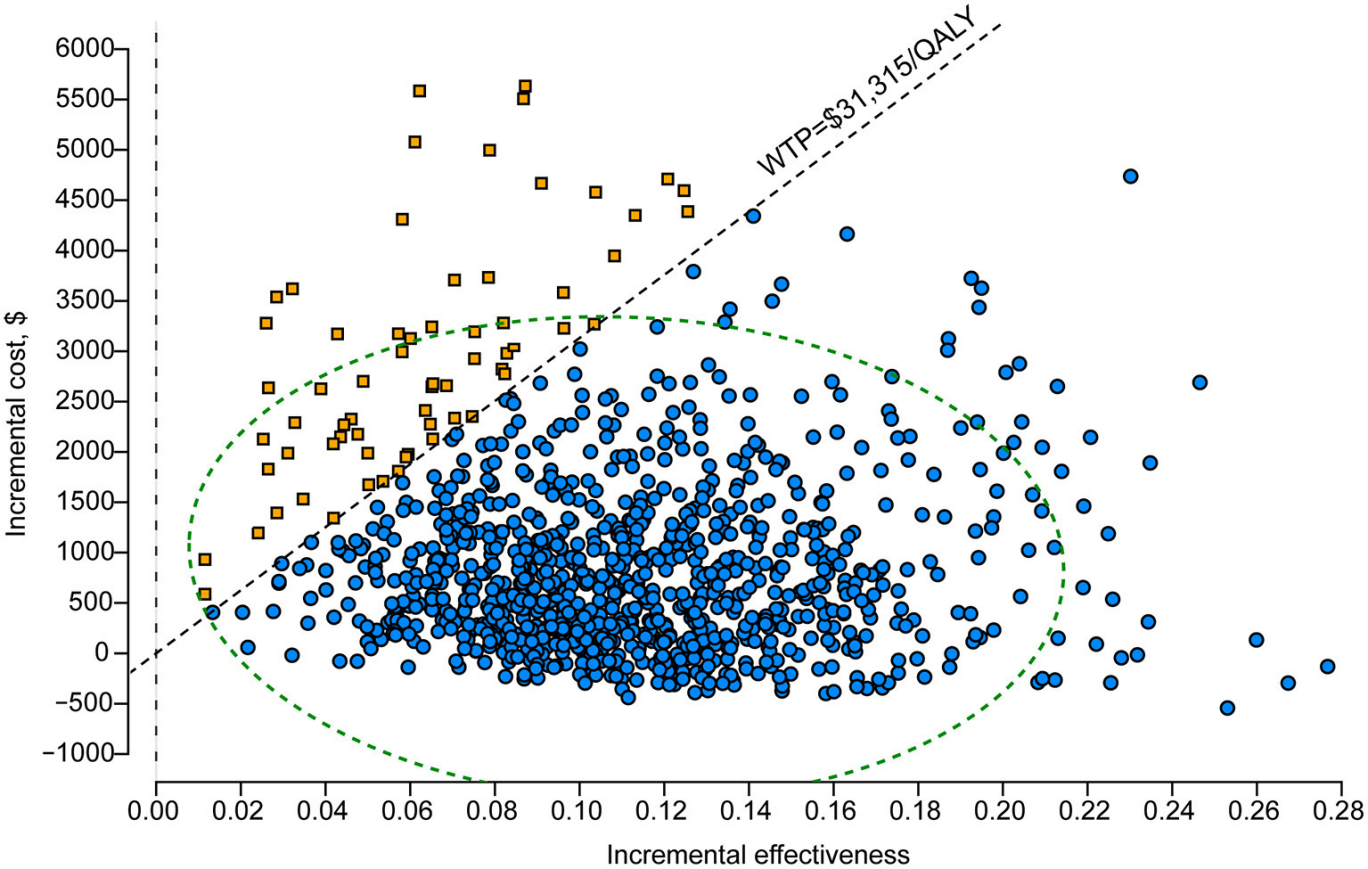
Probabilistic scatter plot of posaconazole vs. first-generation triazoles.

**Figure 4:**
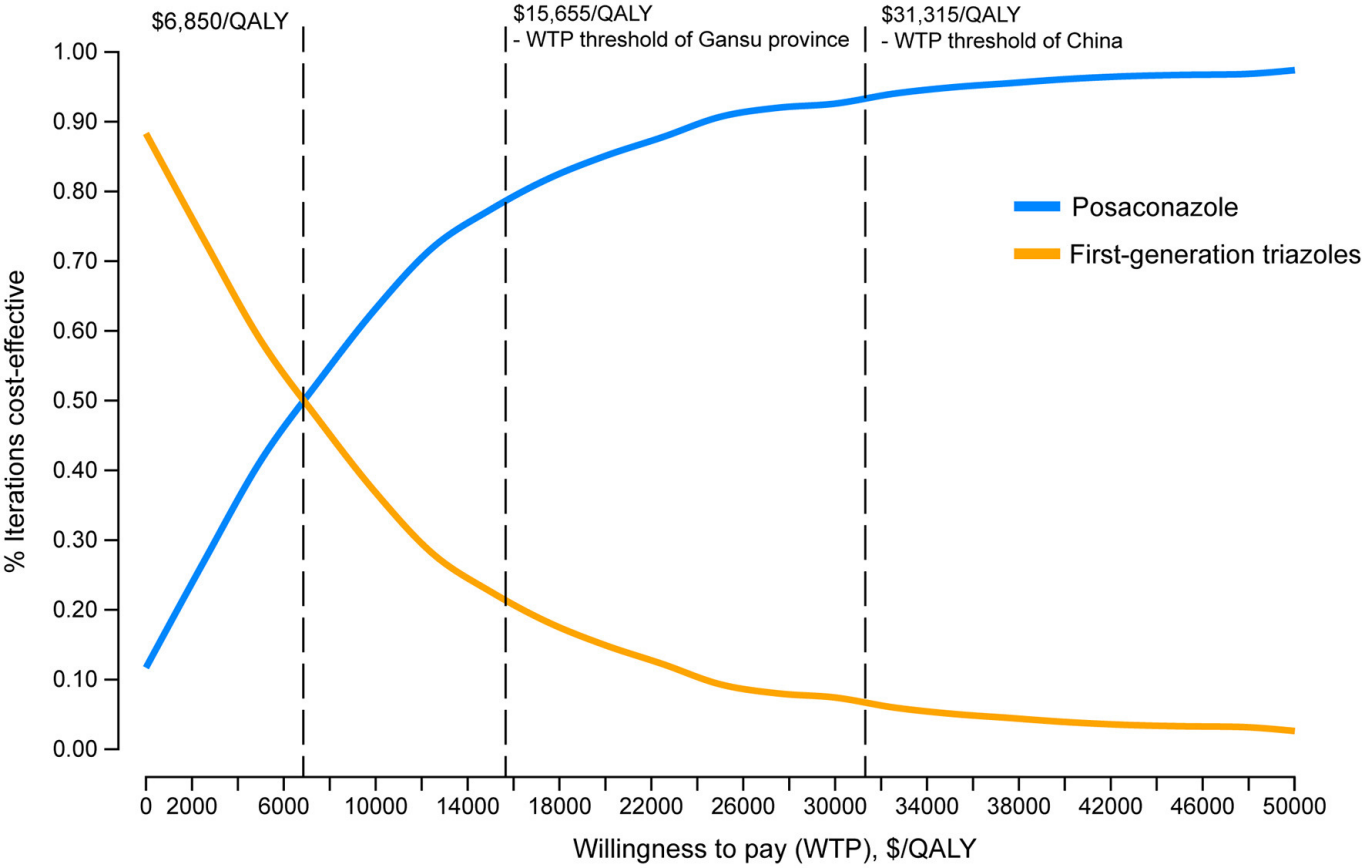
Cost-effectiveness acceptability curve.

One-way DSA showed that the ICER was most sensitive to variations in the probability of IFI with the first-generation triazoles, the cost of posaconazole per day and the duration of posaconazole prophylaxis. However, the ICERs did not exceed the WTP threshold of $31,315/QALY, which was consistent with the conclusion of the base-case analysis (Figure 2). The results of PSA are in line with the base-case analysis, demonstrating that the posaconazole oral suspension had a 93.5% probability of cost-effectiveness at a WTP threshold of $31,315/QALY when compared with the first-generation triazoles (Figure 3). The cost-effectiveness acceptability curve showed that prophylaxis with the posaconazole oral suspension was more likely to be cost-effective than the first-generation triazoles when the threshold of WTP was higher than $6,850/QALY (Figure 4).

### Scenario Analysis

The first-scenario analysis showed that the base-case ICER of $8,784.4/QALY was consistently lower than the thresholds of province-level WTP in all 31 provinces of China. Compared with the first-generation triazoles, the likelihood of the posaconazole oral suspension being cost-effective at the thresholds of province-level WTP ranged from 78.1% (Gansu) to 99.0% (Beijing) (Supplementary Table S1).

In the second-scenario analysis, the ICER increased to $28,641.8/QALY when the oral suspension of posaconazole in the base-case analysis was replaced by the tablet formulation, and this result was still below the national WTP threshold ($31,315/QALY) (Table 2). Compared with the first-generation triazoles, the ICER of the posaconazole tablet formulation was lower than the province-level WTP thresholds in 12 Chinese provinces, indicating that prophylaxis with posaconazole tablets was a cost-effective strategy in these high-income regions (Supplementary Table S1).

## Discussion

The main findings of the study indicated that prophylaxis with a posaconazole oral suspension is highly cost-effective when compared to first-generation triazoles in the prevention of IFIs in high-risk patients with hematological malignancies. Our findings are robust, as shown by the results of both one-way DSA and PSA. Moreover, the conclusions remain valid even in the lowest income province in China according to the province-level scenario analysis, further supporting the economic benefit of prophylaxis with a posaconazole oral suspension in hematological malignancy patients at high risk for IFIs. In addition, the tablet formulation of posaconazole could also be considered a cost-effective antifungal prophylaxis strategy in some high-income provinces in China, although its daily drug costs were much higher than those of the oral suspension formulation.

It should be mentioned that the numerous assumptions applied in the present study were fairly conservative, which may ultimately lead to more reliable conclusions. First, no difference between the two groups in terms of IFI-related mortality and mortality from other causes was assumed in the study; this may have produced an underestimate of the cost-effectiveness of posaconazole, as the latest network meta-analysis of RCTs showed that prophylactic use of posaconazole was associated with a significantly lower all-cause mortality than treatment with fluconazole or itraconazole (11). Second, the lowest drug prices of fluconazole and itraconazole were used in the base-case analysis, which could have led to a less favorable evaluation of posaconazole. Third, the proportion of patients with fluconazole prophylaxis was assumed to be 89% in the first-generation triazoles group, which was higher than that reported in Chinese clinical practice (65%) (1). As fluconazole has much lower daily drug costs than itraconazole in China and there is no significant difference in clinical outcomes between these two first-generation triazoles (11), the assumption of a high proportion of fluconazole use in the model analysis could have led to an underestimation of the cost-effectiveness of posaconazole. Fourth, the management costs of IFIs included medical imaging, antifungal agents and microbiological examinations in the model analysis (20). Of note, costs associated with prolonged hospitalization were not considered as no data were available, which could have led to a less favorable evaluation of posaconazole.

Numerous previous cost-effectiveness analyses addressing the same topic have been conducted during the past dozen years using the same mathematical model, which have been detailed in a pharmacoeconomic review of IFI prophylaxis with posaconazole (23). Regarding the formulations of posaconazole, pharmacoeconomic studies of the oral suspension have been investigated in the USA, Canada, France, Spain, Switzerland, Sweden, Netherlands, Greece, and Hong Kong (24–32), but only a few studies have focused on the tablet formulation (33, 34). The main outcomes of these published studies are summarized in Supplementary Tables S2, 3. These published studies consistently showed that prophylaxis with posaconazole was cost-saving or cost-effective, regardless of its formulations. To the best of our knowledge, this study was the first to assess the cost-effectiveness of two oral formulations of posaconazole vs. first-generation triazoles for IFI prevention in high-risk patients with hematological malignancies from the Chinese perspective. Our findings are consistent with the aforementioned studies conducted outside of the mainland China.

Of note, the formulation of posaconazole used in the pivotal RCT (10) was an oral suspension. This formulation has major drawbacks, including the requirement for multiple-dose administration and the need to be taken with food or a fatty meal (35). To overcome these limitations, a tablet formulation of posaconazole has been developed. Numerous studies have shown that compared to the oral suspension, posaconazole tablets are associated with higher serum drug concentrations and the probability of attaining the target concentration without worsening the safety profile (36–39). As in previous studies (33, 34), the clinical efficacy for IFI prophylaxis with posaconazole tablets was assumed to be equal to that of the oral suspension formulation in our study. Mainly due to the much higher daily drug costs of the tablet formulation of posaconazole compared to the oral suspension formulation, the ICER comparing posaconazole tablets to the first-generation triazoles increased to $28,641.8/QALY. Although the ICER was still lower than the WTP threshold in China, prophylactic use of posaconazole tablets cannot currently be considered a cost-effective regimen in several low-income provinces of China, as the ICER was found to be higher than the WTP thresholds of these provinces. As the prices of fluconazole and itraconazole have significantly decreased since the implementation of the Chinese National Centralized Drug Procurement policy in 2019 (40), additional room for further price reductions of these agents is very limited. Conversely, a substantially reduced drug cost of posaconazole and an improved cost-effective ratio of posaconazole prophylaxis could be anticipated with the emergence of generic competition. Thus, an updated economic evaluation of posaconazole tablets vs. first-generation triazoles for IFI prevention among high-risk patients with hematological malignancies in China is warranted in the future.

Unlike the foreign guidelines, the latest version of the Chinese guidelines recommend posaconazole oral suspension as well as fluconazole and itraconazole for IFI prophylaxis, with no preferential recommendation for one over the other (9). On the basis of previous studies addressing the clinical benefits and our cost-effectiveness results, it is reasonable that the posaconazole oral suspension should be preferentially considered over first-generation triazoles as a first-line prophylaxis regimen in China. In addition, posaconazole tablets could also be recommended for use in select patients who are likely to benefit from the tablet formulation in high-income regions of China.

The present study has several strengths. First, to the best of our knowledge, this study was the first to assess the cost-effectiveness of two oral formulations of posaconazole vs. first-generation triazoles for IFI prevention in high-risk patients with hematological malignancies from the Chinese healthcare perspective. Our findings may be used to provide guidance for decision making and to optimize healthcare resource allocation. Second, imbalanced economic development between different regions of China was well considered in the model analyses. Third, as shown in Supplementary Tables S2, 3, quality of life has not been well considered in most published studies. In the present study, both the quantity and quality of life generated by prophylactic strategies were accounted for, which may accurately reflect the effectiveness of antifungal prophylaxis.

There are also several limitations of the present study. First, the clinical data simulated in the model were primarily from the RCT that was conducted at multiple centers around the world, which might not reflect the actual prophylactic efficacy against IFIs in Chinese populations. Second, the data on the 5-year survival rates of AML and MDS were not specific to Chinese populations due to the lack of relevant studies in China. However, the sensitivity analysis found that the base-case ICER was not sensitive to these factors. Third, our study did not take into account IFI-related outpatient costs. Nonetheless, the impact of these costs on the results of the study was limited, as these costs were estimated to be approximately 3% of the total costs associated with IFI treatment (23).

## Conclusion

In conclusion, our study confirmed that prophylaxis with a posaconazole oral suspension (compared with first-generation triazoles) is a highly cost-effective regimen for preventing IFI among high-risk patients with hematological malignancies in China. Although much higher posaconazole prices are paid for the tablet formulation than for the oral suspension, prophylaxis with posaconazole tablets could also be preferentially considered over that with first-generation triazoles in some high-income regions of China.

## Data Availability Statement

The original contributions presented in the study are included in the article/Supplementary material, further inquiries can be directed to the corresponding authors.

## Ethics Statement

All the data included in this analysis were derived from published literature and public data. No patient- identifiable data were applied or used. Therefore, institutional review board approval was not required.

## Author Contributions

NL and QS contributed to the design of this study. CS, YX, WJ, LW, and YF collected the data. CS, JY, and YX performed the analysis. CS prepared the manuscript. RD helped to revise the manuscript. All authors approved the final version of this study.

## Funding

This work was supported by the Hangzhou Health Science and Technology Planning Project (Grant Number A20200058), the Health Science and Technology Program of Zhejiang Province (Grant Number 2021KY237) and the Hangzhou Agricultural and Social Development Project (Grant Number 20201203B214).

## Conflict of Interest

The authors declare that the research was conducted in the absence of any commercial or financial relationships that could be construed as a potential conflict of interest.

## Publisher's Note

All claims expressed in this article are solely those of the authors and do not necessarily represent those of their affiliated organizations, or those of the publisher, the editors and the reviewers. Any product that may be evaluated in this article, or claim that may be made by its manufacturer, is not guaranteed or endorsed by the publisher.
