# Supplementary material for: Cost-Effectiveness of Posaconazole vs. First-Generation Triazoles for the Prevention of Invasive Fungal Infections Among High-Risk Patients With Hematological Malignancies in China
Source: Front Public Health. 2022 May 17;10:884846. doi: 10.3389/fpubh.2022.884846 (PMC9152267; doi:10.3389/fpubh.2022.884846)
Supplement: Supplementary file 1 [file Table_1.DOCX]

**Table S1**. Scenario analysis results.

| No. | Province | GDP per capita in 2020 (¥) | WTP threshold ($) ^a^ | Posaconazole oral suspension | | Posaconazole tablets | |
| --- | --- | --- | --- | --- | --- | --- | --- |
|  |  |  |  | ICER ^b^ compared with the WTP threshold | Probability of cost-effectiveness (%) | ICER ^b^ compared with the WTP threshold | Probability of cost-effectiveness (%) |
| 1 | Beijing | 164,889 | 71,716 | Lower | 99.0 | Lower | 90.6 |
| 2 | Shanghai | 155,768 | 67,749 | Lower | 98.8 | Lower | 88.5 |
| 3 | Jiangsu | 121,231 | 52,727 | Lower | 98.1 | Lower | 82.3 |
| 4 | Fujian | 105,818 | 46,024 | Lower | 96.8 | Lower | 77.7 |
| 5 | Tianjin | 101,614 | 44,195 | Lower | 96.7 | Lower | 76.4 |
| 6 | Zhejiang | 100,620 | 43,763 | Lower | 96.5 | Lower | 76.2 |
| 7 | Guangdong | 88,210 | 38,366 | Lower | 95.6 | Lower | 71.5 |
| 8 | Chongqing | 78,170 | 33,999 | Lower | 94.7 | Lower | 66.4 |
| 9 | Hubei | 74,440 | 32,376 | Lower | 94.0 | Lower | 63.6 |
| 10 | Shandong | 72,151 | 31,381 | Lower | 93.5 | Lower | 62.5 |
| 11 | Neimenggu | 72,062 | 31,342 | Lower | 93.5 | Lower | 62.5 |
| 12 | Shanxi | 66,292 | 28,833 | Lower | 92.2 | Lower | 59.1 |
| 13 | Anhui | 63,426 | 27,586 | Lower | 92.0 | Higher | 57.0 |
| 14 | Hunan | 62,900 | 27,357 | Lower | 91.9 | Higher | 56.7 |
| 15 | Liaoning | 58,872 | 25,605 | Lower | 91.2 | Higher | 53.1 |
| 16 | Sichuan | 58,126 | 25,281 | Lower | 90.8 | Higher | 52.7 |
| 17 | Jiangxi | 56,871 | 24,735 | Lower | 90.5 | Higher | 51.9 |
| 18 | Henan | 55,435 | 24,111 | Lower | 89.7 | Higher | 51.5 |
| 19 | Hainan | 55,131 | 23,978 | Lower | 89.4 | Higher | 51.4 |
| 20 | Ningxia | 54,528 | 23,716 | Lower | 88.7 | Higher | 50.9 |
| 21 | Xinjiang | 53,593 | 23,309 | Lower | 88.3 | Higher | 49.8 |
| 22 | Xizang | 52,345 | 22,767 | Lower | 87.8 | Higher | 48.5 |
| 23 | Yunnan | 51,975 | 22,606 | Lower | 87.8 | Higher | 48.2 |
| 24 | Qinghai | 50,819 | 22,103 | Lower | 87.4 | Higher | 47.6 |
| 25 | Jilin | 50,800 | 22,095 | Lower | 87.4 | Higher | 47.5 |
| 26 | Shanxi | 50,528 | 21,976 | Lower | 87.1 | Higher | 47.1 |
| 27 | Hebei | 48,564 | 21,122 | Lower | 86.3 | Higher | 45.4 |
| 28 | Guizhou | 46,267 | 20,123 | Lower | 85.3 | Higher | 43.5 |
| 29 | Guangxi | 44,309 | 19,271 | Lower | 84.2 | Higher | 42.2 |
| 30 | Heilongjiang | 42,635 | 18,543 | Lower | 83.5 | Higher | 40.9 |
| 31 | Gansu | 35,995 | 15,655 | Lower | 78.1 | Higher | 34.6 |

^a^ The WTP threshold was set at three times the province-level GDP per capita in 2020.

^b^ Compared to the reference regimen (the first-generation triazoles).

GDP, gross domestic product; ICER, incremental cost-effectiveness ratio; WTP, willingness to pay.
