# Supplementary material for: Cost-Effectiveness of Posaconazole vs. First-Generation Triazoles for the Prevention of Invasive Fungal Infections Among High-Risk Patients With Hematological Malignancies in China
Source: Front Public Health. 2022 May 17;10:884846. doi: 10.3389/fpubh.2022.884846 (PMC9152267; doi:10.3389/fpubh.2022.884846)
Supplement: Supplementary file 2 [file Table_2.DOCX]

**Table S2**. Summary of published studies comparing the cost-effectiveness of posaconazole oral suspension versus first-generation triazoles for the prevention of invasive fungal infections in patients with hematological malignancies.

| Study | County or region | Regimen | Costs | | | LYs (QALYs) | Incremental results ^a^ | | WTP threshold | ICER ($/LY) ^a^ |
| --- | --- | --- | --- | --- | --- | --- | --- | --- | --- | --- |
|  |  |  | Drug | IFI treatment | Total |  | Total costs | LYs (QALYs) |  |  |
| Athanasakis 2013 [24] | Greece | POS oral suspension | €2,115 | €1,291 | €3,406 | 0.744 | €391 | €24,196 | €30,000 | €24,196 |
|  |  | FLU or ITR | €147 | €2,869 | €3,015 | 0.728 | / | / | / | / |
| Grau 2012 [25] | Spain | POS oral suspension | €3,007 | €3,114 | €6,121 | 2.52 | -€1,807 | Dominant | €30,000 | Dominant |
|  |  | FLU or ITR | €450 | €7,478 | €7,928 | 2.43 | / | / | / | / |
| Greiner 2010 [26] | Switzerland | POS oral suspension | CHF4,945 | CHF4,144 | CHF9,098 | 0.744 | -CHF1,118 | Dominant | CHF60,000 | Dominant |
|  |  | FLU or ITR | CHF997 | CHF9,210 | CHF 10207 | 0.728 | / | / | / | / |
| Lundberg 2014 [27] | Sweden | POS oral suspension | SEK26,241 | SEK20,652 | SEK4,6893 | 3.640 (2.621) | -SEK3,124 | Dominant | SEK500,000 | Dominant |
|  |  | FLU or ITR | SEK357 | SEK49,660 | SEK50,017 | 3.536 (2.546) | / | / | / | / |
| Michallet 2011 [28] | France | POS oral suspension | €2,697 | €2,526 | €5,223 | 0.74 | -€859 | Dominant | €30,000 | Dominant |
|  |  | FLU or ITR | €469 | €5,614 | €6,083 | 0.72 | / | / | / | / |
| O'Sullivan 2009 [29] | USA | POS oral suspension | $2,081 | $1,850 | $3,931 | 2.50 | -$575 | Dominant | $50,000 | Dominant |
|  |  | FLU or ITR | $63 | $4,443 | $4,506 | 2.43 | / | / | / | / |
| Stam 2008 [30] | Netherlands | POS oral suspension | €2,693 | €1,781 | €4,414 | 3.70 (2.73) | -€181 | Dominant | €20,000 | Dominant |
|  |  | FLU or ITR | €503 | €4,092 | €4,595 | 3.60 (2.66) | / | / | / | / |
| Tahami 2012 [31] | Canada | POS oral suspension | $4,079 | $2,522 | $6,601 | 2.52 | -$444 | Dominant | $50,000 | Dominant |
|  |  | FLU or ITR | $514 | $6,532 | $7,045 | 2.43 | / | / | / | / |
| Chan 2016 [32] | Hong Kong | POS oral suspension | HK$16,126 | HK$3,503 | HK$19,629 | 2.52 | HK$8,086 | HK$11,6023 | HK$28,5000 | HK$11,6023 |
|  |  | FLU or ITR | HK$3,131 | HK$8,412 | HK$11,543 | 2.45 |  |  |  |  |

^a^ Compared to the reference regimen (the first-generation triazoles).

FLU, fluconazole; ICER, incremental cost-effectiveness ratio; IFI, invasive fungal infection; ITR, itraconazole; LYs, life-years; POS, posaconazole; QALYs, quality-adjusted life-years; WTP, willingness to pay.
