# Supplementary material for: Cost-Effectiveness of Posaconazole vs. First-Generation Triazoles for the Prevention of Invasive Fungal Infections Among High-Risk Patients With Hematological Malignancies in China
Source: Front Public Health. 2022 May 17;10:884846. doi: 10.3389/fpubh.2022.884846 (PMC9152267; doi:10.3389/fpubh.2022.884846)
Supplement: Supplementary file 3 [file Table_3.DOCX]

**Table S3**. Summary of published studies comparing the cost-effectiveness of posaconazole tablets versus first-generation triazoles for the prevention of invasive fungal infections in patients with hematological malignancies.

| Study | County or region | Regimen | Costs | | | LYs (QALYs) | Incremental results ^a^ | | WTP threshold | ICER |
| --- | --- | --- | --- | --- | --- | --- | --- | --- | --- | --- |
|  |  |  | Drug | IFI treatment | Total |  | Total costs | LYs (QALYs) |  |  |
| Cámara 2017 [33] | Spain | POS tablets | €2,691 | €3,215.06 | €5,906.06 | 2.922 | -€1,948.37 | Dominant | €30,000 | Dominant |
|  |  | FLU or ITR | €123.49 | €7,730.94 | €7,854.43 | 2.694 | / | / | / | / |
| Sung 2015 [34] | USA | POS tablet | $4,673 | $2,205 | $6,879 | 2.86 | $1,222 | $6,638 | $50,000 | $6,638 |
|  |  | FLU or ITR | $353 | $5,303 | $5,656 | 2.68 | / | / | / | / |

^a^ Compared to the reference regimen (the first-generation triazoles).

FLU, fluconazole; ICER, incremental cost-effectiveness ratio; IFI, invasive fungal infection; ITR, itraconazole; LYs, life-years; POS, posaconazole; QALYs, quality-adjusted life-years; WTP, willingness to pay.
